# Performance feedback on the quality of care in hospitals performing thrombectomy for ischemic stroke (PERFEQTOS): protocol of a stepped wedge cluster randomized trial

**DOI:** 10.1186/s13063-021-05819-z

**Published:** 2021-12-04

**Authors:** Marzyeh Amini, Sanne J. den Hartog, Nikki van Leeuwen, Frank Eijkenaar, Laurien S. Kuhrij, Lotte J. Stolze, Paul J. Nederkoorn, Hester F. Lingsma, Adriaan C. G. M. van Es, Ido R. van den Wijngaard, Aad van der Lugt, Diederik W. J. Dippel, Bob Roozenbeek, on behalf of the PERFEQTOS Investigators, Bob Roozenbeek, Sanne J. den Hartog, Diederik W. J. Dippel, Aad van der Lugt, Hester F. Lingsma, Nikki van Leeuwen, Laurien S. Kuhrij, Lotte J. Stolze, Paul J. Nederkoorn, Adriaan C. G. M. van Es, Ido R. van den Wijngaard, Paula M. Janssen, Pieter-Jan van Doormaal, Yvo B. W. E. M. Roos, Bart J. Emmer, Suzanne M. Silvis, Wouter Dinkelaar, Michel J. M. Remmers, Douwe L. D. Vos, Rob A. R. Gons, Lonneke S. F. Yo, Julia H. van Tuijl, Hans Kortman, Jelis Boiten, Geert J. Lycklama à Nijeholt, Jurgen R. Piet, Wouter Stomp, Heleen M. den Hertog, H. Zwenneke Flach, Nyika D. Kruyt, Marianne A. A. van Walderveen, Wim H. van Zwam, Robert J. van Oostenbrugge, Jasper M. Martens, Jeannette Hofmeijer, H. Bart van der Worp, Rob T. H. Lo

**Affiliations:** 1 Department of Public Health, Erasmus MC University Medical Center, Rotterdam, The Netherlands; 2 Department of Neurology, Erasmus MC University Medical Center, Rotterdam, The Netherlands; 3 Department of Radiology and Nuclear Medicine, Erasmus MC University Medical Center, Rotterdam, The Netherlands; 4 Erasmus School of Health Policy & Management, Erasmus University Rotterdam, Rotterdam, The Netherlands; 5 Dutch Institute for Clinical Auditing, Leiden, The Netherlands; 6 Department of Neurology, Amsterdam University Medical Centers, location AMC, Amsterdam, The Netherlands; 7 Department of Radiology, Leiden University Medical Center, Leiden, The Netherlands; 8 Department of Neurology, Haaglanden Medical Center, the Hague, The Netherlands; 9 Department of Radiology, Haaglanden Medical Center, the Hague, The Netherlands

**Keywords:** Performance feedback, Endovascular thrombectomy, Stepped wedge cluster randomized trial, Ischemic stroke, Quality of care

## Abstract

**Background:**

Although the provision of performance feedback to healthcare professionals based on data from quality registries is common practice in many fields of medicine, observational studies of its effect on the quality of care have shown mixed results. The objective of this study is to evaluate the effect of performance feedback on the quality of care for acute ischemic stroke.

**Methods:**

PERFEQTOS is a stepped wedge cluster randomized trial in 13 hospitals in the Netherlands providing endovascular thrombectomy for ischemic stroke. The primary outcome is the hospital’s door-to-groin time. The study starts with a 6-month period in which none of the hospitals receives the performance feedback intervention. Subsequently, every 6 months, three or four hospitals are randomized to cross over from the control to the intervention conditions, until all hospitals receive the feedback intervention. The feedback intervention consists of a dashboard with quarterly reports on patient characteristics, structure, process, and outcome indicators related to patients with ischemic stroke treated with endovascular thrombectomy. Hospitals can compare their present performance with their own performance in the past and with other hospitals. The performance feedback is provided to local quality improvement teams in each hospital, who define their own targets on specific indicators and develop performance improvement plans. The impact of the performance feedback and improvement plans will be evaluated by comparing the primary outcome before and after the intervention.

**Discussion:**

This study will provide evidence on the effectiveness of performance feedback to healthcare providers. The results will be actively disseminated through peer-reviewed journals, conference presentations, and various stakeholder engagement activities.

**Trial registration:**

Netherlands Trial Register NL9090. Registered on December 3, 2020

**Supplementary Information:**

The online version contains supplementary material available at 10.1186/s13063-021-05819-z.

## Introduction

### Background and rationale

In 2019, more than 30,000 ischemic stroke patients were admitted to Dutch hospitals and this number is increasing every year [1]. Ischemic stroke is a major cause of death and long-term disability [2]. Randomized trials showed that early endovascular thrombectomy (EVT) substantially improves the 3-month functional outcome with an absolute risk reduction in terms of death or permanent disability of 19.5% [3–5]. With EVT, the neuro-interventionalist advances a catheter through the femoral artery up to the occluded cerebral artery to remove the occlusion of blood clots. The effectiveness of EVT is highly time-dependent. Every hour delay in the initiation of EVT results in death or permanent disability in 1 out of every 19 patients [6]. Several trials and observational studies noted an association between an efficient workflow to achieve fast recanalization and a stronger treatment effect of EVT resulting in better clinical outcomes [7–11]. Therefore, having the right infrastructure and an efficient process of care is of utmost importance to be able to treat every patient as fast as possible.

Performance feedback has been defined as “a summary of clinical performance on a specific indicator, e.g. process measure, with benchmarking against performance of other providers over a specified period of time with or without recommendations for action” [12–15]. Providing performance feedback regarding process indicators to healthcare professionals has become quite common in healthcare [12, 16]. However, performance feedback has no firm empirical basis and consensus on how this feedback is best to be provided is lacking [12]. A Cochrane systematic review of 140 studies on the effectiveness of audit and performance feedback on the quality of care reported a median 4.3% absolute improvement in patient outcomes (interquartile range 0.5–16%) [12]. However, effects tend to be very heterogeneous, and success factors for the design and delivery of effective performance feedback have not been identified. Understanding heterogeneity of effect has been limited in part by the lack of appropriate design of the feedback interventions and the absence of a quantitative evaluation of effectiveness [17–20]. The aim of this study is to evaluate the effect of providing performance feedback to healthcare providers in individual EVT hospitals to improve the quality of stroke care.

### Objectives

Since stroke logistics and time metrics vary considerably between centers and within centers, and are strongly associated with outcome [21], this study aims to assess the extent to which performance feedback to healthcare providers in individual hospitals providing EVT for ischemic stroke, resulting in action plans and targets based on this feedback, reduces time from arrival at the hospital to initiation of EVT and thereby improves the quality of care.

## Methods

### Trial design

**Fig. 1:**
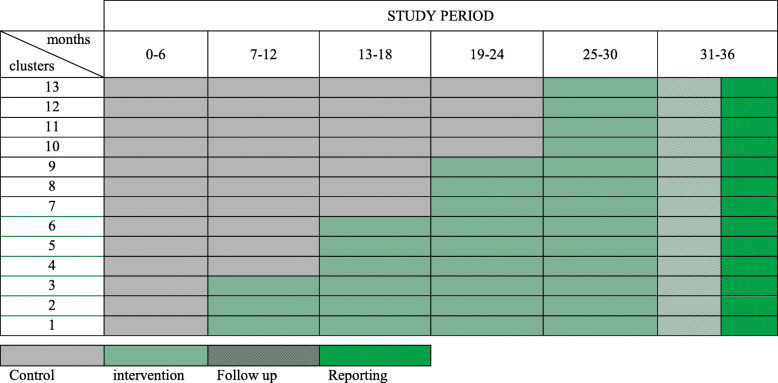
SPIRIT figure. Schematic visualization of stepped wedge cluster randomized trial control and intervention allocation, follow-up, and reporting of the study

We designed a stepped wedge cluster randomized trial (CRT) of performance feedback on the quality of care in hospitals performing EVT for ischemic stroke (PERFEQTOS) to determine the impact of a performance feedback intervention accompanying hospital-specific action plans and improvement targets. This is a specific form of CRT in which initially all clusters (in this study: hospitals) serve as controls. The intervention is rolled out sequentially but randomly at different time points, such that at the end of the study all clusters have crossed over to the intervention condition (Fig. 1) [22]. In the present study, the design includes an initial 6-month period in which none of the hospitals receives performance feedback. Subsequently, every 6 months, three or four randomly chosen hospitals will cross over from control to intervention conditions, which implies a total study duration of 30 months given that 13 hospitals participate. The Standard Protocol Items: Recommendation for Interventional Trials (SPIRIT) checklist [23] is shown in Additional file 1.

### Study setting and participants

**Fig. 2:**
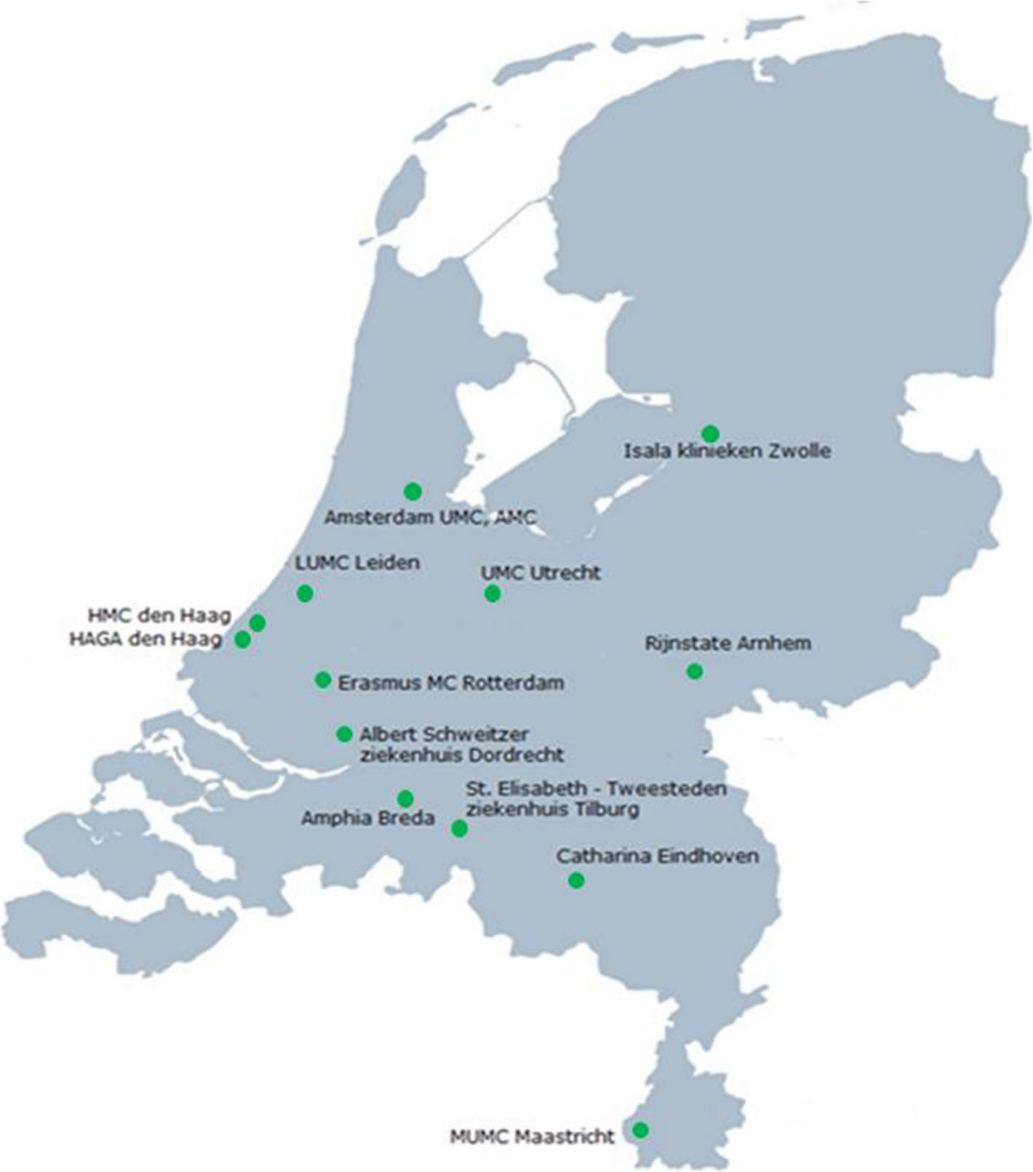
Specialized EVT hospitals in the Netherlands which participate in this study

The study will be carried out in specialized neuro-intervention hospitals performing EVT for ischemic stroke. In the Netherlands, EVT is concentrated in 17 such specialized hospitals [24]. All EVT hospitals are invited to participate in this trial with no specific inclusion or exclusion criteria. Thirteen out of these 17 hospitals agreed to participate in the study (Fig. 2). The participating hospitals include all admitted adult patients with acute ischemic stroke who underwent EVT.

### Randomization and intervention allocation

Randomization will be performed according to a lottery method of sampling by the Study Coordinator in the presence of the Principal Investigator and an independent observer and will be videotaped. Once the hospitals provided consent to participate in the study, each enrolled hospital will start in the control condition. Every 6 months, three to four hospitals will be randomized to cross over to the intervention condition. All other hospitals stay in the control condition. This procedure continues every 6 months until all hospitals are crossed over to the intervention condition. Participating hospitals will be blinded to the allocation sequence, and those hospitals not yet in the intervention condition will not be aware of the time at which they will cross over to the intervention condition. When a hospital is crossed over to the intervention, this is communicated to all participating hospitals.

### Intervention

#### Dashboard design

**Fig. 3:**
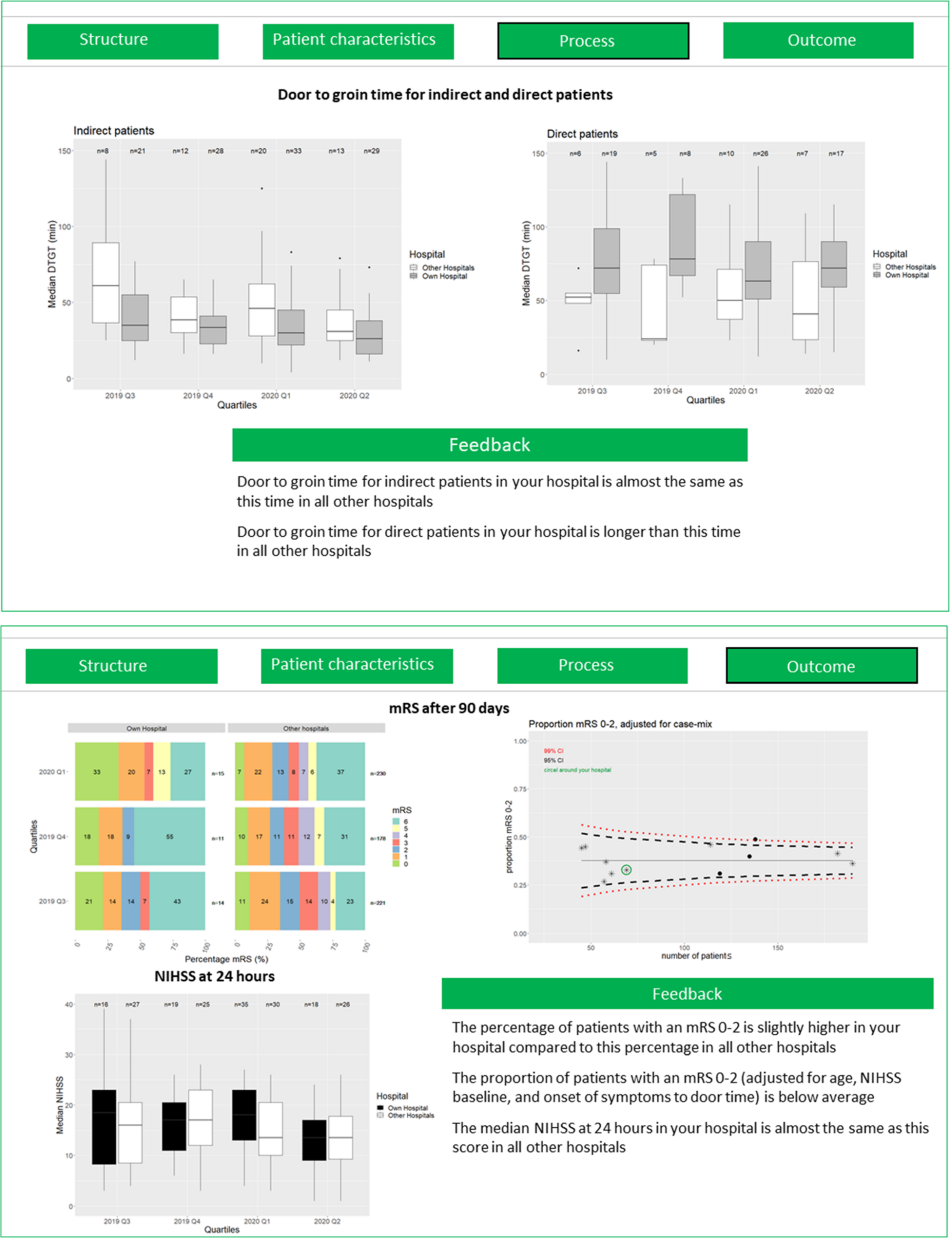
Screenshots of the PERFEQTOS dashboard (generated from anonymous data)

When it comes to performance feedback, it is necessary to visualize and communicate the information content in such a way that care providers can use this information for improvement and to maximally reduce the risk of misinterpretation of the results [25]. With this in mind, we developed a dashboard containing quality of care measures per hospital, benchmarked against the average/median performance of the other hospitals for the same time period as well as their own performance over time. Prior to designing this dashboard, we reviewed the general empirical literature on dashboard design [25–32]. Based on the insights from previous studies, we composed a dashboard containing a compositional qualitative and quantitative (graphical display with textual explanation) visualization of quality of care data. Specifically, the PERFEQTOS dashboard provides an overview of results on quality of care indicators and patient characteristics (Fig. 3). The performance feedback is presented in a way that clearly highlights the key message (i.e., improvement is recommended or not), limiting the amount of extra information to increase actionability while still allowing the recipient hospital to view more detailed comparative information.

### Indicator measurement for the dashboard

Evaluation of healthcare providers’ quality performance is commonly done using a combination of structure, process, and outcome indicators (Table 1) [33, 34]. Our dashboard follows that approach.

**Table 1:** Quality of care indicators included in the dashboard

Quality indicators	Type	Definition
Center volume	Structure	The total number of patients treated with EVT in each individual hospital
Number of transferred patients	Structure	Whether patient transferred from another hospital to an EVT hospital
Door-to-needle time	Process	Time from arrival at the emergency department of EVT hospital to IVT initiation
Door-to-groin time	Process	Time from arrival at the emergency department of EVT hospital to groin puncture
eTICI	Outcome	Thrombolysis in cerebral infarction scale to assess intracranial reperfusion, ranging from 0 (no reperfusion) to 3 (full reperfusion)
NIHSS at 24 h	Outcome	The neurological deficit ranged between 0 (normal function) and 42 (completely impaired)
mRS at 3 months	Outcome	Functional outcome score of mRS evaluated 3 months after EVT treatment ranged between 0 (no symptom) and 6 (death)

*eTICI* extended thrombolysis in cerebral infarction, *NIHSS* National Institute of Health Stroke Scale, *mRS* modified Rankin Scale, *EVT* endovascular thrombectomy, *IVT* intravenous thrombolytics

Structure indicators are the total number of patients treated with EVT in each hospital (henceforth referred to as “center volume”), the number of direct (i.e., non-transferred) and transferred stroke patients from other hospitals, and completeness of data.

Process indicators are time from arrival at the emergency department of the intervention hospital to start of the intravenous thrombolytics (IVT) administration (door-to-needle time) for non-transferred patients and time from arrival at the emergency department of the intervention hospital to initiation of EVT (door-to-groin time) for all patients. In case a patient has an ischemic stroke while already admitted to the hospital, then the arrival time is defined as the time the neurologist first assessed the patient. We stratified the indicators by whether patients are transferred or non-transferred from another hospital.

Outcome indicators are post-EVT reperfusion grade of the Extended Thrombolysis in Cerebral Infarction (eTICI) score, ranging from 0 (no reperfusion) to 3 (full reperfusion), and the National Institute of Health Stroke Scale (NIHSS) score at 24 h (± 12 h), which quantifies the neurological deficit caused by a stroke (range 0–42). In addition, the modified Rankin Scale (mRS) score is used as a measure of patients’ functional outcome after acute ischemic stroke, ranging from 0 (no symptoms) to 6 (death). The mRS score is assessed 3 months after admission. Since the outcome is strongly associated with baseline patient characteristics [35], patients’ age, sex, NIHSS score on arrival, location of the proximal intracranial occlusion, and time from onset to arrival at the intervention hospital (onset-to-door time) are also collected. Stroke onset is defined as the time point when stroke symptoms were first noticed by the patient or an observer. In cases the time of first symptoms is unknown, onset is defined as the moment the patient was last seen well. Additionally, we adjusted the outcomes per hospital for differences in these baseline characteristics.

### Strategy for quality improvement

**Fig. 4:**
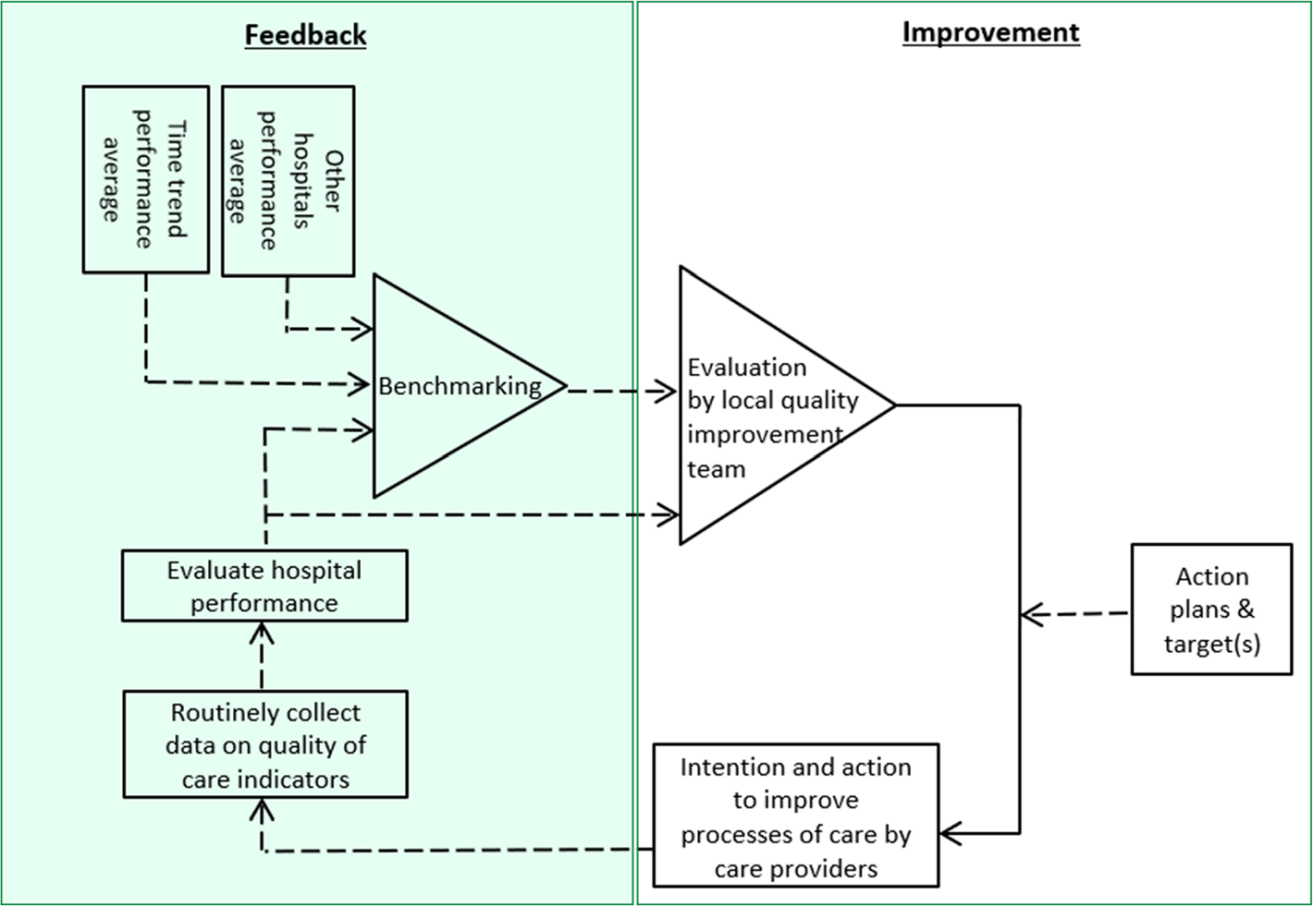
The performance feedback cycle to improve each hospital’s performance. Adapted from Carver and Scheier’s Control Theory [36]

The performance improvement cycle is depicted in Fig. 4 to reflect the procedures through which hospitals aim to improve their clinical performance. Each hospital randomized to the intervention will install a local quality improvement team. This team consists at least of a neurologist, an interventionalist, a resident in neurology, and a (stroke) nurse. It can be expanded with representatives of other relevant disciplines. Each quality improvement team is trained by one of the PERFEQTOS investigators to explain the dashboard functionalities, interpretation of data, and how action plans can be developed. Quality improvement teams of hospitals in the intervention cluster periods receive quarterly performance feedback reports, which they can use to set improvement targets pertaining to specific indicators and develop performance improvement plans to achieve those targets. The assumption is that if a center’s performance is below that of the comparator, an improvement target will be set accordingly, and subsequent improvement actions would be aimed at reaching the target and remove the discrepancy [16, 36, 37]. The impact of these actions can then be evaluated based on the next feedback report(s).

In addition, during workshops organized every 6 months for those hospitals randomized to the intervention, both the best performing hospital and most improved hospital share their best practices with the other hospitals.

### Data collection and management

Data on performance indicators and patient characteristics are routinely collected in each hospital and reported to the Dutch Acute Stroke Audit (DASA) from the Dutch Institute of Clinical Auditing (DICA). DASA is the main prospective clinical auditing tool for stroke in The Netherlands since 2014, with the aim to assure the quality of patient care and to aid in improving outcomes [38]. DICA is an independent organization, founded by medical specialists, that facilitates national audits for various medical professions, including the DASA. Hospitals are free to decide who carries out the data registration (e.g., nurses, data managers, neurologists), but the final responsible person is a neurologist. Medical Research Data Management (MRDM) processor, a trusted third party, is involved to pseudonymize the data to comply with privacy legislation. Hospitals can provide the collected data to MRDM in three ways. First, an online survey through a secured web environment is available for hospitals to record the data. Second, hospitals can provide the data in batches, i.e., data files in which large amounts of data can be transferred directly to MRDM. Third, to minimize the registration burden, some hospitals took the initiative to implement data linkage, i.e., extracting the data from their individual electronic patient health record to be automatically forwarded to MRDM [38]. Participating hospitals approved with a written agreement that MRDM send pseudo raw data about the patients treated in their hospital to the PERFEQTOS Study Coordinator at Erasmus MC. The Study Coordinator then aggregates the data into hospital-level medians and percentages. Data collection continues throughout the study, so each hospital contributes to both control and intervention cluster periods, which will be compared. Next, the aggregated data are summarized in a report and fed back via the dashboard. All hospitals randomized to the intervention group will receive a performance feedback report on a quarterly basis and will start and re-iterate the performance improvement cycle (Fig. 4).

### Study outcomes

The primary outcome is door-to-groin time. Secondary outcomes are door-to-needle time, eTICI score, NIHSS at 24 h, and mRS at 3 months (Table 1). These outcomes are collected for every individual patient (Table 1).

### Power calculation

The power calculation was based on mean differences in door-to-groin time as a primary outcome. We used a parametric power estimation methodology for stepped wedge designs put forward by Hemming and Girling [39] in the Stata function stepped wedge, derived from Hussy and Hughes [40]. We assumed 13 EVT centers were randomized in four clusters per time step (three clusters of three hospitals and one cluster of four hospitals) that treated an average of 30 patients per center per time period of 3 months [21]. We used a mean door-to-groin time of 77 min (standard deviation 47 min) and *ICC* 0.37, both obtained from the MR CLEAN Registry data [21, 24]. Assuming a significance level of 5%, this would result in 88% power to detect a clinically relevant reduction of door-to-groin time of 10 min.

### Statistical methods

The effect of the intervention on the primary and secondary outcomes will be assessed by comparing median/percentage outcomes between control and intervention cluster periods and also in the time trends of intervention cluster periods using a non-parametric Kruskal-Wallis test and Pearson’s chi-square statistic, respectively.

Generalized linear mixed modeling at the individual patient level will be used for analyzing the effect of the performance feedback intervention on the primary outcome (door-to-groin time). This will allow us to appropriately account for the different observation cluster periods (both control and intervention) and the hierarchical structure of the data [40]. The model will contain fixed effects for intervention (yes/no), calendar time (month) to account for autonomous time trends, patient characteristics (i.e., patients’ age, sex, NIHSS score at arrival, location of the proximal intracranial occlusion, and onset-to-door time) [40], and a random effect for the hospital. This model enables us to estimate the variance of the outcome of interest (door-to-groin time) at the hospital level (inter-cluster variation) and individual level (intra-cluster variation), which in turn enables us to estimate the total effect of the performance feedback intervention. The increase in the variance due to the clustering will be quantified by the variance inflation factor. In sensitivity analyses, we will estimate the impact of the duration of exposure to the performance feedback intervention, the effect of moving from control to the intervention condition, and/or interaction of both factors on the primary outcome [41].

Multivariable normal models will be used for imputation of missing values, using available data on patient characteristics, structure, and care processes.

### Oversight and data monitoring

The trial Executive Committee consists of one Principal Investigator, one junior Study Coordinator, two researchers affiliated to DICA, three vascular neurologists, one neuro-interventionalist, one radiologist, and two trial methodologists. The Trial Steering Committee is the main decision-making body. It consists of members of the Executive Committee and all local Principal Investigators. The Steering Committee meets at least once a year.

All incoming data are reviewed by the Study Coordinator at the central trial office. All pseudo data from DASA are stored on the Erasmus MC’s secure server, which is only accessible by the Study Coordinator and Principal Investigator.

This study will be conducted in accordance with the principles of Good Clinical Practice, the Dutch Agreement on Medical Treatment Act (WGBO), and the European General Data Protection Regulation. All patients will receive the best medical treatment according to national and local guidelines and current insights. We use data collection exclusively for improving quality of care purposes. To protect patients’ privacy, the trusted third party MRDM processes the data on behalf of the healthcare providers in such a way that the Study Coordinator receives only pseudonymized patient data, meaning that personal data has been processed in such a way that they can no longer be linked to a specific person. These additional data are stored separately, and technical and organizational measures are taken to ensure that personal data can never be linked to a specific person.

This trial does focus on (performance feedback about) the treatment of acute ischemic stroke. No reason to assume that the ancillary or post-trial care varies between participating hospitals or is influenced by the intervention tested in this trial. Therefore, we do not expect this to confound the trial’s results.

### Dissemination policy

The Executive Committee forms the Writing Committee for the trial. Publications will be made on behalf of all investigators. The main study results will be disseminated via publication in an international peer-reviewed journal and presentation at international conferences for health provider specialists. Representatives of the participating hospitals will be given the opportunity to comment on the manuscript and participate as co-authors. We plan to disseminate the results of the planned secondary analyses on the feasibility and effectiveness of performance feedback in one or more separate papers.

## Discussion

PERFEQTOS is a stepped wedge cluster randomized trial about the effect of performance feedback on the quality of acute ischemic stroke care. This performance feedback consists of a multifaceted intervention, including the implementation of a quality indicator dashboard, quality improvement teams, and performance improvement plans. The primary outcome is a process measure (door-to-groin time) which is actionable and strongly associated with clinical outcomes [6].

Previously, it has been noticed that a stepped wedge CRT design is more efficient when the intra-cluster correlation (*ICC*) is moderate or high (*ICC* ≥ 0.1) [42, 43]. Generally, *ICC* tends to be higher for process indicators in comparison to clinical outcomes, which is related to the fact that processes can be more easily influenced by providers than outcomes [43]. Therefore, since our main focus is on improving care processes through performance feedback, a stepped wedge CRT design is an efficient design for this study. Another strength of the stepped wedge CRT design is that it provides an opportunity to measure the effect of duration of exposure to the intervention as well as of underlying temporal changes. In order to take advantage of this strength, the longitudinal time intervals should be sufficiently broad [40]. We hypothesize 6 months is long enough to achieve an effect based on treatment outcome measurements (e.g., functional outcome at 3 months after stroke). This design helps us also to tackle difficulties of implementation of the intervention at all hospitals at once. Additionally, the intervention effect in all hospitals can be evaluated with different time exposures (6–18 months).

The (design of the) current study has some limitations. First, given that our intervention is multifaceted, it may be difficult to disentangle the relative impact of the different aspects of the intervention. However, an intervention with a combination of different strategies is more effective than approaches using a single intervention [44]. Second, the quality improvement policies of hospitals in the control phase are heterogeneous and this may influence the effect of our intervention. To facilitate the interpretation of our results, we will use semi-annual questionnaires to obtain insight into the quality improvement policies of the hospitals in the control arm. Third, outcome assessment is not blinded in our trial. However, the primary outcome (door-to-groin time) is an objective measure and therefore unlikely to bias the trial results. The assessment of the secondary outcomes NIHSS at 24 h and mRS at 3 months is not blinded as well, but because these outcomes are routinely collected through a structured algorithm, we expect the risk of bias to be limited.

Findings from this study will provide insight into the feasibility and effectiveness of structured performance feedback in reducing the time to treatment of patients with ischemic stroke treated with endovascular thrombectomy and, thereby, in improving patient outcomes. If the expected results are realized, the developed method for performance feedback is ready for wider implementation on a national and international level and can be adapted for use in other diseases.

## Trial status

Protocol version number: 2, August 2020. PERFEQTOS has started in January 2020, and the anticipated study duration is 30 months.

## Supplementary Information

**Additional file 1.** SPIRIT Checklist.

## Data Availability

Data cannot be made available, as no patient approval has been obtained for sharing coded data. However, syntax files and output of statistical analyses will be made available upon request after the trial is finalized.

## Abbreviations

CRT: Cluster randomized trial
DASA: Dutch Acute Stroke Audit
DICA: Dutch Institute of Clinical Auditing
eTICI: Extended thrombolysis in cerebral infarction
EVT: Endovascular thrombectomy
ICC: Intra-cluster correlation coefficient
IVT: Intravenous thrombolytics
MRDM: Medical Research Data Management
mRS: Modified Rankin Scale
NIHSS: National Institute of Health Stroke Scale
